# LRRC1 Promotes Angiogenesis Through Regulating AKT/GSK3β/β-Catenin/VEGFA Signaling Pathway in Hepatocellular Carcinoma

**DOI:** 10.3390/cells14231919

**Published:** 2025-12-03

**Authors:** Huanfei Liu, Zhentao Liu, Peitong Xie, Zihan Liu, Yaqing Zhang, Lanxiao Cao, Ning Shang, Mei Chen, Huixing Feng, Xiaowen Guan, Guifu Dai

**Affiliations:** School of Life Sciences, Zhengzhou University, Zhengzhou 450001, China

**Keywords:** tumor angiogenesis, hepatocellular carcinoma, LRRC1, AKT/GSK3β, VEGFA

## Abstract

**Highlights:**

**What are the main findings?**

- LRRC1 promotes hepatocellular carcinoma (HCC) angiogenesis by activating the AKT/GSK3β/β-catenin/VEGFA signaling pathway.
- LRRC1 enhances PDK1 expression by acting as a scaffold to recruit USP7 to deubiquitinate PDK1, thereby leading to increased AKT1 phosphorylation.

**What are the implication of the main finding?**

- LRRC1 is identified as a novel potential target for anti-angiogenic therapy in HCC.
- The elucidated LRRC1-VEGFA axis provides a theoretical basis for developing combination treatment strategies.

**Abstract:**

Tumor angiogenesis plays a crucial role in the progression of hepatocellular carcinoma (HCC), serving as a key process for metastasis and invasion. Leucine-rich repeat-containing 1 (LRRC1) has been reported to be abnormally upregulated in HCC. Nevertheless, the specific mechanism through which LRRC1 affects HCC is poorly understood. In our study, analysis of public datasets reveals a positive correlation between LRRC1 and VEGFA, which drives us to hypothesize the linkage between LRRC1 and tumor angiogenesis. Herein, we aimed to explore the role of LRRC1 in HCC angiogenesis and the involved mechanisms. In vitro, LRRC1 overexpression significantly increased the mRNA, protein, and secretory levels of VEGFA and promoted tumor-induced migration, invasion, and tube formation of HUVECs. Conversely, these effects were suppressed by the knockdown of LRRC1. In vivo, LRRC1 promoted the formation of new blood vessels in the chick embryo chorioallantois membrane, together with tumor growth and angiogenesis in xenograft mice. Further mechanism studies showed that LRRC1 enhances PDK1 stability by promoting its deubiquitination via USP7, thereby increasing AKT1 phosphorylation levels and activating the AKT/GSK3β/β-catenin/VEGFA signaling pathway, ultimately accelerating tumor angiogenesis in HCC. These findings demonstrated a novel role of LRRC1 in tumor angiogenesis, opening up new avenues for future research and treatment development.

## 1. Introduction

The inducing vasculature is considered to be one of the hallmarks of solid cancer progression [1]. Among these, angiogenesis, the formation of new blood vessels from pre-existing ones, is an essential process for tumor growth and development [2]. Tumor cells promote the formation of new blood vessels by secreting pro-angiogenic factors like vascular endothelial growth factor (VEGF), and in turn, the new blood vessels provide oxygen and nutrients to further support the growth, invasion, and metastasis of cancer cells [3,4]. In general, increased tumor angiogenesis and high expression of pro-angiogenic factors are associated with the advanced stage and poor prognosis in multiple solid cancers, including hepatocellular carcinoma (HCC) [5], non-small cell lung cancer (NSCLC) [6], colorectal cancer [7], etc. Among these cancers, HCC, as one of the most common malignant tumors worldwide, is often diagnosed at an advanced stage and is particularly severe in China [8,9]. The growth and metastasis of HCC are highly dependent on aberrant vascular supply and vessel co-option, which together constitute a key characteristic of HCC progression [10]. Hence, a better understanding of the mechanisms responsible for tumor angiogenesis can be useful for developing effective approaches to attenuate HCC progression.

Among the broad spectrum of pro-angiogenic factors, VEGFA is a key molecule to promote angiogenesis in the tumor microenvironment and exerts control over the proliferation, migration, sprouting, and vessel elongation through activating VEGF receptor 2 (VEGFR2) that is primarily expressed on endothelial cells [11,12]. Thus, targeting VEGF signaling has been accepted as a therapeutic approach to disrupt tumor vessel formation and inhibit tumor growth over the past decades. VEGF inhibitors, such as bevacizumab, sorafenib, and aflibercept, significantly attenuate angiogenesis and have been used in the treatment of multiple cancers [13]. In particular, bevacizumab, a monoclonal antibody that specifically targets VEGFA, has been widely used as the first-line therapy for patients with advanced-stage HCC in the past years [14]. Nevertheless, like most targeted drugs, VEGF antagonists also encounter both primary and secondary resistance after treatments over time, limiting the effectiveness of their clinical application [15,16]. In this context, it is pivotal to explore novel molecules involving the regulatory function of VEGFA, which will provide additional targeted therapies combined with the existing strategies to overcome drug resistance and inhibit HCC angiogenesis.

Leucine-rich repeat-containing 1 (LRRC1), also known as LANO, is a member of the leucine-rich repeat and PDZ domain (LAP) family. It has been reported to play an important role in different biological processes, including cell polarity, proliferation, and differentiation [17,18,19]. For example, high LRRC1 levels in acute myeloid leukemia can promote the proliferation and glycolysis by upregulating the β-catenin/c-Myc axis [20]. Moreover, a recent bioanalysis of multiple datasets from the Gene Expression Omnibus (GEO) database demonstrated that LRRC1 was upregulated in HCC tissues and was significantly correlated with the overall survival of HCC patients [21,22]. However, the specific role of LRRC1 in the progression of HCC remains unclear. Coincidentally, the preliminary analysis of public datasets indicates a positive correlation between LRRC1 and VEGFA, prompting us to hypothesize and verify the linkage between LRRC1 and tumor angiogenesis.

Therefore, this study aims to explore the biological function of LRRC1 in HCC angiogenesis and the underlying mechanisms involved. Through a series of experiments, including studies in xenograft mouse models and in vitro assays, we identify that LRRC1 is a potent tumor angiogenesis regulator. Mechanistically, LRRC1 promotes PDK1 expression by acting as a scaffold to recruit USP7 to deubiquitinate PDK1 and, in turn, activates the downstream AKT/GSK3β/β-Catenin/VEGFA signaling pathway. These findings provide direct experimental evidence for the crucial role of LRRC1 in tumor angiogenesis and further offer a deeper insight into understanding the regulatory network of HCC.

## 2. Materials and Methods

### 2.1. Cells and Reagents

HepG2 and HCC-LM3 cell lines were obtained from Conservation Genetics CAS Kunming Cell Bank (Kunming, China) and the Cell Bank of the Chinese Academy of Sciences (Shanghai, China), respectively. HUVECs were kindly provided by Professor Xiukun Lin of the Institute of Oceanology, Chinese Academy of Sciences. Cell lines, including HCC-LM3 and HepG2, were maintained in high-glucose DMEM; HUVECs were maintained in RPMI-1640 medium. The DMEM and RPMI 1640 medium were supplemented with 10% FBS, and 1 ×Penicillin-Streptomycin Solution. All cells were cultured in an incubator at 37 °C and 5% CO_2_.

The primary antibodies, anti-LRRC1 (10128-2-AP), anti-VEGFA (19003-1-AP), anti-p-AKT1 (66444-1-Ig), anti-β-catenin (66379-1-Ig), anti-CD31 (11265-1-AP), anti-GFP (66002-1-Ig), anti-PDK1 (18262-1-AP), anti-USP7 (66514-1-Ig), ubiquitin (10201-2-AP), and anti-GAPDH (60004-1-Ig), were purchased from Proteintech (Wuhan, China). Anti-AKT1 (ET1609-47), anti-p-GSK3β (ET1607-60), and anti-GSK3β (ET1607-71) were purchased from HUABIO (Hangzhou, China). The secondary antibodies: goat anti-mouse IgG (31430) and goat anti-rabbit IgG (31460), were purchased from Thermo Fisher.

### 2.2. Patients and Tissue Specimens

The tissue chips were purchased from Shanghai Outdo Biotech (Shanghai, China), which contained HCC tissues and the corresponding noncancerous tissues from 25 patients, including 2 cases with intrahepatic cholangiocarcinoma.

### 2.3. Cell Transfection

For the LRRC1 overexpression experiment, the pcDNA3.1-LRRC1-EGFP vector (LRRC1-OV) or pcDNA3.1-EGFP vector (EV) obtained from Fenghui Biotech (Shanghai, China) was transfected into low endogenous LRRC1-expressing HCC-LM3 cells with Lipo8000™ as per the guidelines provided by the manufacturer. G418 (700 μg/mL) was used to screen stable cell lines after the cells were transduced with transfection reagents and continued to be cultured for 48 h. The infection efficiency was detected by Western blot and qRT-PCR.

For the LRRC1 knockdown experiment, the plasmids of pGPU6-GFP-Neo- LRRC1-Homo611 (shLRRC1) or negative control shRNA (shNC) obtained from Gene Pharma (Shanghai, China) were transfected into high-endogenous LRRC1-expressing HepG2 cells with Lipo8000™ as per the guidelines provided by the manufacturer. The screening method for stable cell lines was the same as described above.

The siRNAs targeting AKT1 were synthesized by TsingKe Biotech (Zhengzhou, China) and were transfected into HCC-LM3 cells with Lipo8000™. The sequences for the sh-LRRC1 and si-AKT1 were listed in Supplementary Table S1.

### 2.4. Preparation of Tumor Conditional Medium

HCC cells were divided into four groups in 6-well plates: HepG2+shNC, HepG2+shLRRC1, HCC-LM3+EV, HCC-LM3+LRRC1-OV groups and cultured for 48 h before collecting the supernatant, which we called tumor conditional medium (CM). The CM was harvested and centrifuged at 2000 rpm for 10 min to remove the cell fragments and stored at −80 °C. The secreted levels of VEGFA in CM collected from the LRRC1-OV, EV, shLRRC1, or shNC cells were detected by microplate reader using a commercial kit (Jianglai, Shanghai, China).

### 2.5. Invasion Assay

To assess the invasion ability of HUVECs, the Matrigel (354234, BD Biosciences, Franklin Lakes, NJ, USA) was diluted with DMEM medium in a ratio of 1:7 and then added to each upper chamber (50 μL/well). The prepared chambers were incubated at 37 °C for 3 h for the following experiments. In total, 2  ×  10^5^ cells in 200 μL of serum-free medium were seeded into the upper chamber covered by Matrigel, while 600 μL CM collected from the LRRC1-OV, EV, shLRRC1, or shNC cells containing 10% FBS was added to the lower chamber. After incubating for 24 h, the non-migrating cells in the upper chamber were removed, and the cells in the lower chamber were fixed with methanol at RT for 20 min, followed by dyeing with crystal violet solution for 10 min. The images were captured using a microscope in 5 random visible fields in every well, and cell number was counted using Image-J software (version 1.52a).

### 2.6. Scratch Assay

The migration capability of HUVECs was analyzed using a scratch assay. Briefly, 2 × 10^4^ HUVECs per 200 μL culture medium were seeded into a 96-well plate and cultured for 24 h, followed by creating a scratch using a 200 μL pipette tip. Then the cells were treated with CM collected from the LRRC1-OV, EV, shLRRC1, or shNC cells and allowed to migrate for 24 h. The scratch area was documented using an inverted microscope (SOPTOP, Ningbo, China), and the scratch areas of the cells were measured at 0 h and 24 h using ImageJ software (version 1.52a). The relative migration rate was calculated as follows: (scratch area at 0 h-scratch area at 24 h)/scratch area at 0 h × 100%.

### 2.7. Tube Formation Assay

A 96-well plate coated with Matrigel (50 μL/well) was incubated at 37 °C for 30 min. Then, HUVECs (2 × 10^4^) were suspended in 100 μL of CM collected from the LRRC1-OV, EV, shLRRC1, or shNC cells and placed on the pre-coated 96-well plate. After incubation at 37 °C for another 6 h, images were taken by a microscope. The tube nodes and total length of each group were counted using ImageJ software (version 1.52a).

### 2.8. Chick Chorioallantois Membrane Assay

The chick chorioallantois membrane (CAM) model was used to assess the effect of CM collected from the LRRC1-OV, EV, shLRRC1, or shNC cells on angiogenesis in vivo. On the 7th day of embryo development, the eggs were opened with forceps on the air chamber side, and the CAM of each egg was exposed by removing the shell membrane. Then the sterilized filter paper was gently placed on the chorionic membrane and treated with CM collected from HCC cells with stable overexpression or knockdown of LRRC1. Subsequently, the eggs were sealed and incubated for 2 days at 38 °C and 80% relative humidity. The CAM was fixed with methanol for 20 min, then gently removed and placed on a slide. Each membrane was randomly photographed with a microscope for 5 fields, and the microvascular density was calculated using Image-Pro Plus (version 6.0.0.260).

### 2.9. Animal Experiment

Male BALB/c-nude mice (3–4 weeks old, weighing 14–15 g) were obtained from Vital River Laboratory Animal Technology (Beijing, China). The mice were housed in a standard environment with a 12 h cycle of light and darkness. All the experiments were approved by the Experimental Animal Ethics Committee of Zhengzhou University (ZZUIRB 2022-126).

The subcutaneous neoplasia mouse model was established through injecting LRRC1-OV cells (LRRC1-OV group) or EV cells (EV group) (0.1 mL, 7 × 10^6^ per animal) into the right axilla of nude mice. The body weight and tumor size of the mice were measured every two or three days. According to the animal welfare policy, the mice were sacrificed when the maximal tumor length reached about 1.5 cm, then the subcutaneous tumors were removed and fixed in 4% paraformaldehyde for 24 h.

### 2.10. Immunohistochemistry Staining

Paraffin-embedded liver tissue samples were cut into 3 µm thick slices, deparaffinized with xylene and graded ethanol, and washed with 1× PBS. The slices were placed in freshly prepared 3% hydrogen peroxide and incubated for 20 min away from light to remove peroxidase. Then the slides were heated in antigen retrieval solution for 10 min and cooled with running water. The area of tissues was circled with a special immunohistochemical pen and incubated with 2% BSA for 40 min; after removing the blocking agent, the diluted anti-LRRC1, anti-VEGFA, anti-β-catenin, and anti-p-Smad2/3 primary antibodies were added and incubated overnight at 4 °C. Then, the secondary antibodies were added and incubated for 1 h at RT. The slides were stained with 3, 3′-diaminobenzidine (DAB) and terminated with running water, then dehydrated in xylene and sealed with neutral gum. We randomly selected 5 visual fields from each slice to analyze the immunohistochemistry (IHC) score and the number of nuclear positive cells. The intensity score was classified as 0 (negative), 1 (weak), 2 (moderate), and 3 (strong), and the density score was indicated as 1 (0–25%), 2 (26–50%), 3 (51–75%), and 4 (76–100%). Then, the total IHC scores were generated by multiplying these two scores.

### 2.11. Total RNA Extraction and qRT-PCR Assay

Total RNA was extracted from cultured cells by Trizol reagent (Thermo Fisher Scientific, Waltham, MA, USA) and quantified by Nanodrop 2000 (Thermo Fisher Scientific, Waltham, MA, USA). Approximately 1.0 µg of total RNA was used for the reverse transcription with BeyoRT™II First Strand cDNA Synthesis Kit (Beyotime, Shanghai, China), and qRT-PCR was conducted using SYBR Green qPCR mix (Beyotime, Shanghai, China) in a QuantStudio 5 system (ABI, Thermo Fisher Scientific, Waltham, MA, USA). Relative quantification regarding gene expression was analyzed by the 2^−ΔΔCt^ methodology, using GAPDH as an internal control. The primer sequences are shown in Supplementary Table S2.

### 2.12. Protein Extraction and Western Blot Assay

Cells were washed twice with cold PBS and lysed in RIPA buffer (Beyotime, Shanghai, China) containing 1% proteinase inhibitor and 1% phosphatase inhibitor (Beyotime, Shanghai, China). The protein concentrations were detected through BCA protein assay kit (CWBIO, Taizhou, China). Equimolar amounts of protein were separated by 10% sodium dodecylsulfate-polyacrylamide gel electrophoresis (SDS-PAGE) and transferred to NC membranes. Membranes were blocked with Tris-buffered saline containing 5% nonfat dry milk for 1 h at RT and then incubated with the primary antibody diluted with western blot antibodies dilution (Beyotime, Shanghai, China) overnight at 4 °C. Then, the secondary antibodies were added and incubated for 1 h at RT. Finally, the NC membrane was detected by using Hypersensitive ECL chemiluminescence kit (Beyotime, Shanghai, China) and visualized by a chemiluminescence imager (C300, Azure Biosystems, California, USA). GAPDH was used as the internal reference, and the range of band grayscale values was analyzed using ImageJ software (version 1.52a).

### 2.13. Co-Immunoprecipitation (Co-IP) Assay

Cells were washed twice with cold PBS and lysed in IP lysis buffer (Beyotime, Shanghai, China) containing 1% proteinase inhibitor and 1% phosphatase inhibitor (Beyotime, Shanghai, China) for 30 min at 4 °C. After centrifugation at 12,000× *g* for 10 min, the protein concentrations were detected through BCA protein assay kit (CWBIO, Taizhou, China), and equal amounts of protein were incubated with Protein A/G PLUS-Agarose beads (Beyotime, Shanghai, China) and different antibodies overnight at 4 °C. Then the immune complex was washed five times with washing buffer and detected by WB.

### 2.14. Statistical Analysis

All results were presented as mean ± standard deviation (SD). All experiments with statistical analysis have been repeated at least three times. Data analysis was conducted utilizing GraphPad Prism software (version 8.0.1). Student’s *t*-test was used to compare two groups, and one-way ANOVA was used when comparing three or more groups. *p* < 0.05 was considered statistically significant.

## 3. Results

### 3.1. LRRC1 Expression Levels Are Elevated in HCC Tissues and Positively Correlated with the Expression Levels of VEGFA

**Figure 1:**
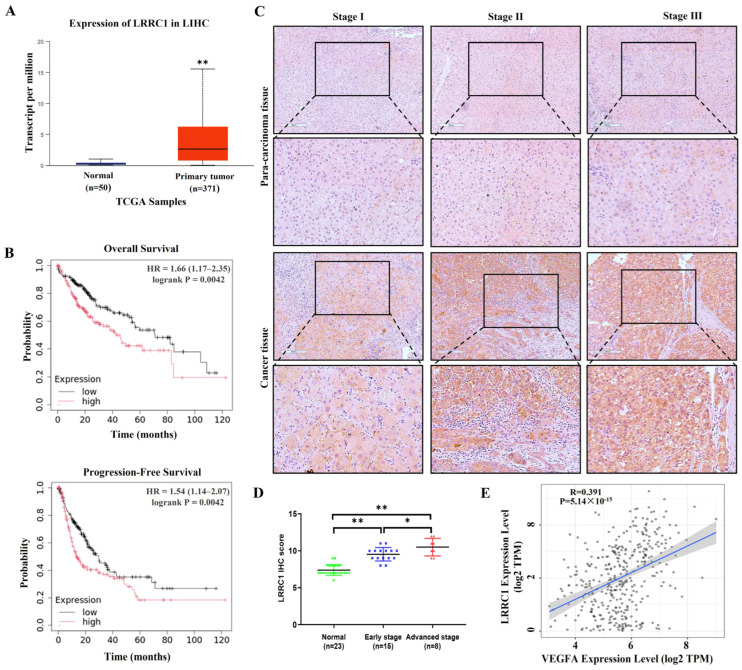
LRRC1 levels are higher in HCC tissues than in the normal tissues and positively correlated with the VEGFA expression levels. (**A**) The mRNA levels of LRRC1 in normal liver tissues (*n* = 50) and primary tumor tissues (*n* = 371) in the TCGA database. (**B**) KM survival analysis of HCC patients with high LRRC1 expression and low LRRC1 expression. (**C**) Representative images of immunohistochemistry (IHC) staining showing LRRC1 expression in tumor tissues and para-carcinoma tissues derived from clinical HCC tissue microarray. Scale bar: 200 μm. (**D**) Scatter plots showing the distribution of the IHC scores for LRRC1 expression in different stages of HCC tissues (early stage: stages I and II, *n* = 15; advanced stage: stages III and IV, *n* = 8). (**E**) The correlation analysis between LRRC1 and VEGFA expressions in HCC using TCGA database (R = 0.391, *p* < 5.14 × 10^−15^). Data represented as the mean ± SD (* *p* < 0.05, ** *p* < 0.01).

To investigate the role of LRRC1 in HCC development, firstly, we analyzed HCC datasets from The Cancer Genome Atlas (TCGA) database using the online UALCAN (https://ualcan.path.uab.edu/analysis.html accessed on 5 November 2024) tool. The mRNA levels of LRRC1 were significantly upregulated in HCC tissues compared with those in non-tumor tissues (*p* < 0.01, Figure 1A). Kaplan–Meier survival analysis revealed that patients with high LRRC1 expression had poor overall survival (OS) and progression-free survival (PFS) (*p* < 0.01, Figure 1B). Additionally, the remarkably elevated protein levels of LRRC1 in HCC tissues compared with those in the para-carcinoma tissues were validated (*p* < 0.01) through analyzing LRRC1 expression levels in tumor tissue microarray (Figure 1C,D). As is widely accepted, high angiogenic dependence is one of the key features of HCC [23]. Intriguingly, as shown in Figure 1E, the analysis with TIMER2.0 (http://timer.cistrome.org/) confirmed a positive correlation of LRRC1 with VEGFA (R = 0.391, *p* < 5.14 × 10^−15^), a potent pro-angiogenetic factor in HCC [24], suggesting high LRRC1 expression may contribute to angiogenesis in HCC.

### 3.2. LRRC1 Positively Regulates the Expression of VEGFA

**Figure 2:**
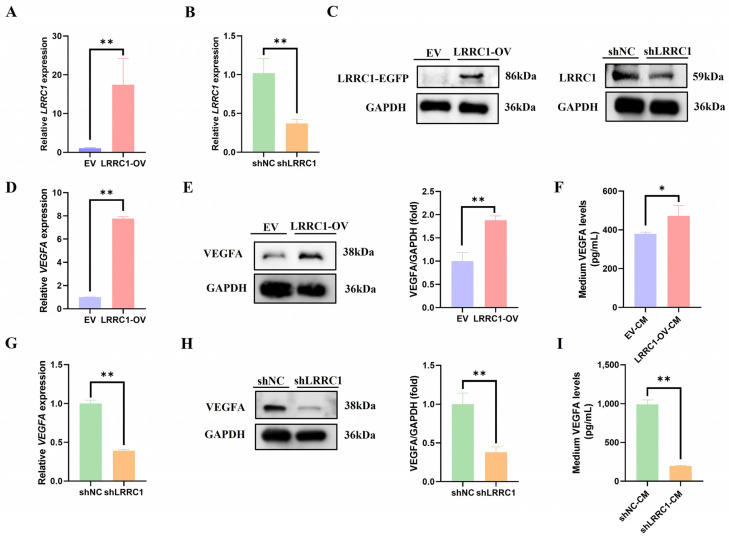
LRRC1 positively regulates the expression of VEGFA in vitro. (**A**,**B**) The LRRC1 mRNA levels in the LRRC1-overexpressing HCC-LM3 cells (LRRC1-OV), LRRC1-knockdown HepG2 cells (shLRRC1), and the corresponding control cells (EV or shNC). (**C**) Representative western blot images of LRRC1 in LRRC1-OV, EV, shLRRC1, and shNC cells. (**D**) The mRNA levels of VEGFA in LRRC1-OV cells and EV cells. (**E**) The protein levels of VEGFA in LRRC1-OV cells and EV cells. (**F**) The levels of secreted VEGFA in tumor-conditioned medium (CM) from LRRC1-OV cells and EV cells. CM was harvested after incubation for 48 h. (**G**) The mRNA levels of VEGFA in shLRRC1 cells and shNC cells. (**H**) The protein levels of VEGFA in shLRRC1 cells and shNC cells. (**I**) The levels of secreted VEGFA in CM from shLRRC1 cells and shNC cells. CM was harvested after incubation for 48 h. Data represented as the mean ± SD (* *p* < 0.05, ** *p* < 0.01).

To elucidate the potential biological function of LRRC1 in HCC angiogenesis, we first established stable LRRC1-overexpressing HCC-LM3 cells (with low endogenous expression) and LRRC1-knockdown HepG2 cells (with high endogenous expression) (Figure S1 and Figure 2A–C). We then examined whether gain or loss of LRRC1 altered the expression level of VEGFA. As presented in Figure 2D–F, both intracellular and secreted VEGFA levels were significantly increased in LRRC1-overexpressing cells compared with the corresponding control group (*p* < 0.05); while those in LRRC1-knockdown cells were obviously reduced (*p* < 0.01, Figure 2G–I). Together, these findings reveal that LRRC1 positively regulates the expression of VEGFA in HCC cells.

### 3.3. Tumor-Induced Angiogenesis Is Promoted by LRRC1 Through Regulating VEGFA Secretion

**Figure 3:**
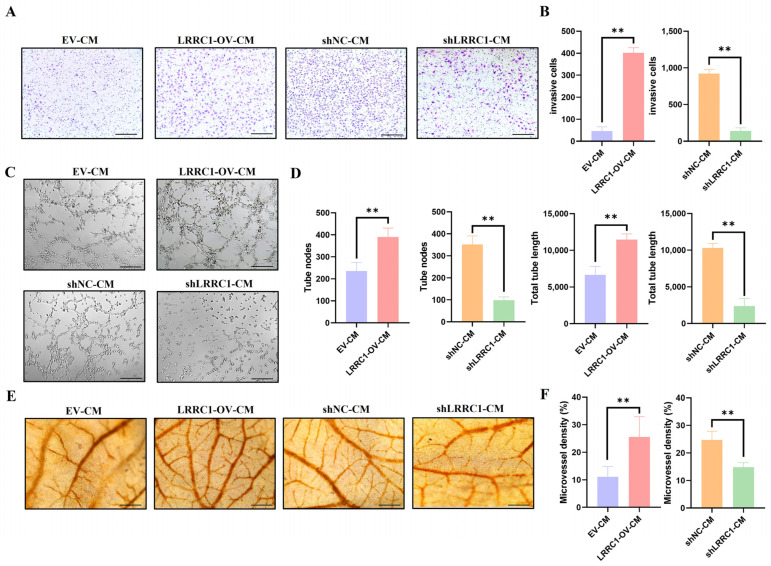
LRRC1 promotes tumor-induced angiogenesis through increasing the secretion of VEGFA. (**A**,**B**) Representative images (**A**) and quantification (**B**) of invasive cells of HUVECs treated with tumor conditioned medium (CM) collected respectively from the LRRC1-overexpressing HCC-LM3 cells (LRRC1-OV), corresponding control cells (EV), LRRC1-knockdown HepG2 cells (shLRRC1), and the corresponding control cells (shNC) for 24 h (*n* = 3). Scale bars, 200 µm. (**C**,**D**) Representative images (**C**) and quantification (**D**) of tube formation of HUVECs treated with CM from the indicated cells for 6 h (*n* = 3). Scale bars, 200 µm. (**E**,**F**) Representative images (**E**) and quantification (**F**) of chicken chorioallantois membrane (CAM) after being treated with CM from indicated cells for 48 h (*n* = 8). Scale bars, 400 µm. Data represented as the mean ± SD (** *p* < 0.01).

To explore the function of LRRC1 in promoting tumor-induced angiogenesis, the conditioned medium (CM) collected from different HCC cell lines, representing the VEGFA secreted by cells with different LRRC1-expression levels (Figure 2F,I), was used to treat HUVECs. It was found that compared with the cells treated with CM from control cells, HUVECs treated with CM from LRRC1-overexpressing cells presented stronger abilities of invasion (*p* < 0.01, Figure 3A,B), migration (*p* < 0.01, Figure S2A,B), and tube formation (*p* < 0.01, Figure 3C,D). In contrast, CM from LRRC1-knockdown cells markedly inhibited these abilities (*p* < 0.01). Furthermore, the chick chorioallantois membrane (CAM) assay also showed that the number of new blood vessels was obviously reduced after LRRC1 knockdown (*p* < 0.01), and overexpression of LRRC1 could promote the formation of new blood vessels (*p* < 0.01, Figure 3E,F). Taken together, these data further confirm that LRRC1 drives tumor-induced angiogenesis via soluble mediators in the medium, indicating a pro-angiogenic role of LRRC1 in HCC.

### 3.4. Overexpression of LRRC1 Promotes Tumor Growth and Angiogenesis in HCC-LM3 Xenograft Mice

**Figure 4:**
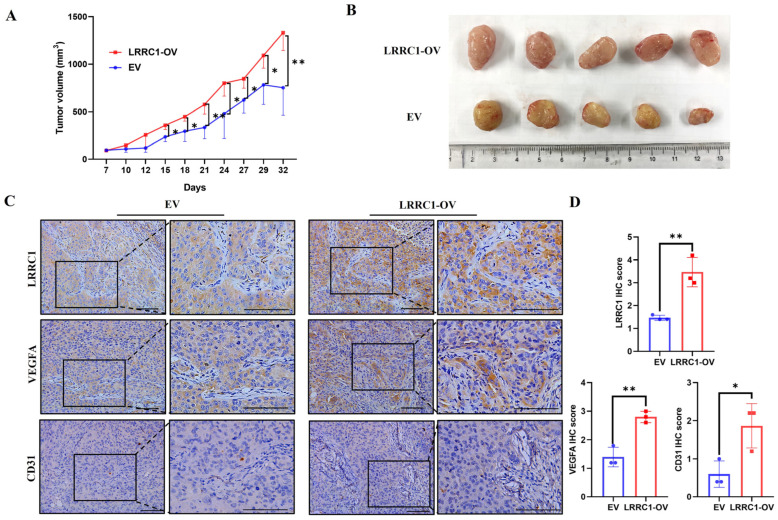
Overexpression of LRRC1 promotes tumor growth and angiogenesis in HCC-LM3 xenograft mice. (**A**) Volume of indicated tumor at different time points was shown using time-course line plot (*n* = 5). (**B**) Images of xenografted tumors of nude mice in the LRRC1-OV group and EV group after 32-day subcutaneous transplantation (*n* = 5). (**C**,**D**) Representative images (**C**) and quantification (**D**) of IHC analysis of LRRC1, VEGFA, and CD31 levels in xenografted tumor tissues from EV and LRRC1-OV groups (*n* = 3). Scale bars, 100 µm. Data represented as the mean ± SD (* *p* < 0.05, ** *p* < 0.01).

To further explore the function of LRRC1 in driving tumor growth and angiogenesis, a subcutaneous xenograft mouse model was established. As shown in Figure 4A,B, the volumes of tumors in the LRRC1-OV group were significantly higher than those in the EV group from 15 days after cell injection till the end of the experiment (*p* < 0.05), supporting the important role of LRRC1 in the development of HCC. Interestingly, it was found that the tumor tissues from the LRRC1-OV group had more abundant blood vessels than those from the EV group (Figure 4B). In congruence with in vitro results, the IHC staining analysis showed that protein expressions of VEGFA and CD31 in the tumor tissues of xenografted mice from the LRRC1-OV group were remarkably increased compared with those from the EV group, indicating that LRRC1 plays a critical role in the promotion of tumor growth and tumor angiogenesis of HCC in vivo (*p* < 0.05, Figure 4C,D).

### 3.5. LRRC1 Increases VEGFA Expression via AKT/GSK3β/β-Catenin Signaling Pathway

**Figure 5:**
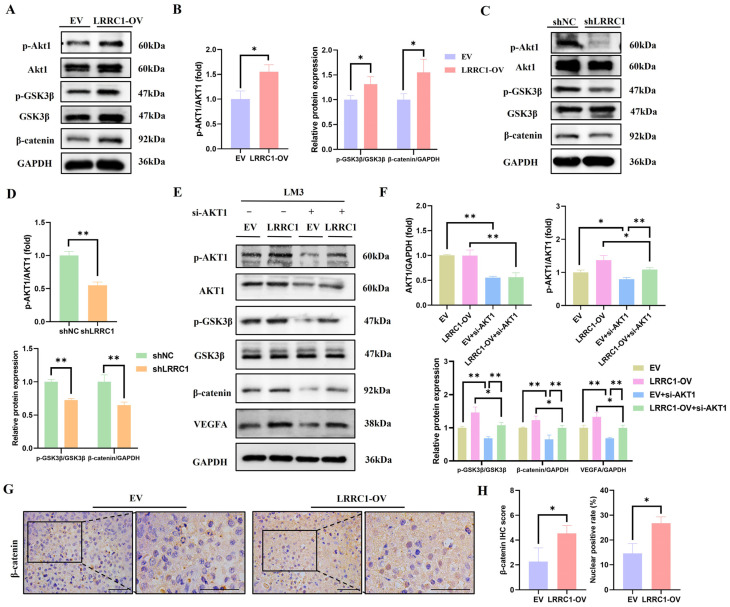
LRRC1 activates AKT/GSK3β/β-catenin axis. (**A**,**B**) Representative western blot images (**A**) and quantification (**B**) of p-AKT1, p-GSK3β, and β-catenin in LRRC1-overexpressing cells (LRRC1-OV) and the corresponding control cells (EV). (**C**,**D**) Representative western blot images (**C**) and quantification (**D**) of p-AKT1, p-GSK3β, and β-catenin in LRRC1-knockdown cells (shLRRC1) and the corresponding control cells (shNC). (**E**,**F**) Western blot analysis of the expressions of p-AKT1, AKT1, p-GSK3β, β-catenin, and VEGFA in LRRC1-overexpressing HCC-LM3 cells after transfection with the si-AKT1. (**G**) Representative images of IHC staining of the expression of β-catenin in xenografted tumor tissues from EV group and LRRC1-OV group (*n* = 3). Scale bars, 50 µm. (**H**) Statistical analysis of expressions and nuclear positive rates of β-catenin in xenografted tumor tissues from indicated groups. Data represented as the mean ± SD (* *p* < 0.05, ** *p* < 0.01).

The AKT pathway acts as a central regulator of angiogenesis in HCC by directly regulating its key downstream effector VEGFA [25,26,27]. Notably, AKT1 could weaken the suppressive effect of GSK3β on β-catenin through phosphorylating GSK3β, thereby promoting the expression of VEGFA [28]. Therefore, we speculated whether LRRC1 could influence the VEGFA expression through the AKT/GSK3β/β-catenin pathway. The western blot results showed that the protein levels of p-AKT1, p-GSK3β, and β-catenin were significantly increased in the LRRC1-overexpressing cells (*p* < 0.05, Figure 5A,B). Accordingly, IHC analysis demonstrated that the levels of p-AKT1 and p-GSK3β were higher in tumor tissues from mice in the LRRC1-OV group than those in the EV group (*p* < 0.01, Figure S3); in contrast, those in LRRC1-knockdown cells were obviously suppressed (*p* < 0.01), compared with the corresponding control cells (Figure 5C,D).

To determine whether AKT1 mediates the regulation of VEGFA by LRRC1, the small interfering RNAs (siRNAs) were used to suppress the expression of AKT1. As shown in Figure 5E,F, the results of western blot analysis showed that the expression levels of p-AKT1, GSK3β, β-catenin, and VEGFA were significantly reduced after inhibiting AKT1 (*p* < 0.05), while this reduction was attenuated by the overexpression of LRRC1 (*p* < 0.05). Furthermore, we observed increased nuclear localization of β-catenin in tumor tissues from the LRRC1-OV group compared with the EV group (*p* < 0.05, Figure 5G,H). As nuclear β-catenin is known to promote VEGFA expression and drive angiogenesis [29], these data suggest that LRRC1 promotes VEGFA through the AKT/GSK3β/β-catenin axis. Above all, we conclude that the AKT/GSK3β/β-catenin signaling pathway participates in the regulatory effect of LRRC1 on VEGFA.

### 3.6. LRRC1 Serves as a “Scaffold” to Recruit USP7 for Deubiquitylation of PDK1

**Figure 6:**
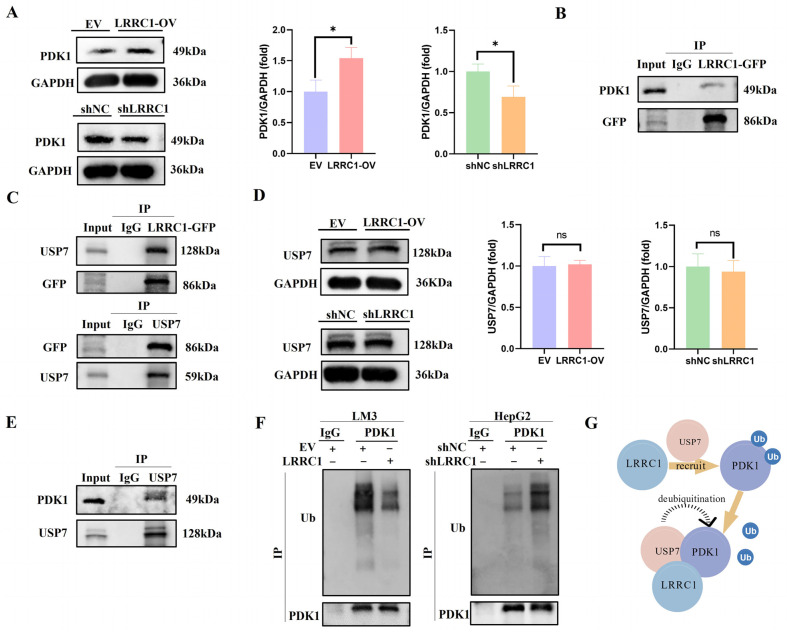
LRRC1-mediated PDK1 deubiquitylation mechanism. (**A**) Representative western blot images and quantification of PDK1 in LRRC1-overexpressing HCC-LM3 cells (LRRC1-OV), corresponding control cells (EV), LRRC1-knockdown HepG2 cells (shLRRC1), and the corresponding control cells (shNC). (**B**) Immunoprecipitation experiments verifying that LRRC1 and PDK1 can physically interact. (**C**) Immunoprecipitation experiments verifying that LRRC1 and USP7 can physically interact. (**D**) Representative western blot images and quantification of USP7 in LRRC1-OV, EV, shLRRC1, and shNC cells. (**E**) Immunoprecipitation experiments verifying that USP7 and PDK1 can physically interact. (**F**) Ubiquitination assay to analyze the effect of LRRC1 on PDK1 ubiquitination-mediated. (**G**) Schematic diagram of LRRC1 serves as a “scaffold” to recruit USP7 for deubiquitylation of PDK1 degradation. Data represented as the mean ± SD (* *p* < 0.05).

As a primary upstream kinase that phosphorylates AKT, PDK1 forms a critical signaling axis with AKT. The activation of the PDK1-AKT axis is a well-established hallmark governing cell proliferation, survival, drug resistance, and metabolism in various cancers [30]. We therefore hypothesized that LRRC1 regulates AKT signaling via modulation of PDK1. As shown in Figure 6A, LRRC1 overexpression significantly increased PDK1 protein levels, while LRRC1 knockdown decreased them (*p* < 0.05). Furthermore, Co-IP assays confirmed a physical interaction between LRRC1 and PDK1 in HCC cells (Figure 6B). Notably, mass spectrometry data from a previous study by Jean-Paul Borg suggested a potential interaction between LRRC1 and USP7, a critical deubiquitinating enzyme [31]. Our Co-IP results confirmed that LRRC1 indeed binds to USP7 (Figure 6C), although it was found that LRRC1 overexpression or knockdown did not alter USP7 protein levels (Figure 6D). Interestingly, as a previous study mentioned, USP7 could deubiquitinate PDK1 in a K48-linked manner [32]. Consistent with this, Co-IP analysis in our study revealed that USP7 also endogenously binds to PDK1 in HCC cells (Figure 6E). These intriguing findings suggest that LRRC1, USP7, and PDK1 bind to each other to form a ternary complex. We thus propose that LRRC1 may serve as a scaffold protein that recruits USP7 to deubiquitinate PDK1, thereby protecting it from ubiquitin-mediated degradation. Consistent with this model, Co-IP analysis showed that LRRC1 knockdown promoted the ubiquitination level of PDK1, whereas overexpression of LRRC1 attenuated this phenomenon (Figure 6F). Together, these results demonstrate that LRRC1 enhances PDK1 expression by promoting its deubiquitylation via USP7 (Figure 6G), thereby leading to increased AKT phosphorylation in HCC.

## 4. Discussion

As a key process required for metastasis and invasion, tumor angiogenesis plays a pivotal role in the progression of cancer [33]. In the last decade, anti-angiogenic agents targeting VEGF signaling have been widely used as an effective non-surgical treatment for HCC patients. However, like most targeted drugs, VEGF antagonists also encounter both primary and secondary resistance after treatments over time, limiting the effectiveness of their clinical application. The mechanisms underlying resistance to anti-angiogenic therapy are multifaceted, such as VEGF-independent sprouting angiogenesis driven by pro-angiogenic growth factors and vessel co-option, where tumors utilize pre-existing host vessels [34,35]. Evidence confirms that tumors can utilize vessel co-option as an alternative to angiogenesis, facilitating acquired resistance to anti-angiogenic therapy in HCC [35]. Collectively, these mechanisms underscore the molecular complexity of HCC progression and contribute to the severe drug resistance that currently limits the utility of these agents [36]. Therefore, a deeper exploration of these molecular mechanisms and the identification of novel pro-angiogenic factors are essential for developing more effective therapeutic strategies. In our present work, we found the important role of LRRC1 in HCC angiogenesis for the first time. We also revealed that the expression of LRRC1 in HCC tissues was higher than that in adjacent tissues and closely related to the stage of HCC. Consistent with our findings, previous studies have demonstrated the oncogenic role of LRRC1 in HCC, NSCLC, and cholangiocarcinoma. For example, it has been reported that miR-193a promotes apoptosis in cisplatin-resistant NSCLC cells by downregulating LRRC1 [37], indicating that high LRRC1 expression contributes to cisplatin resistance in NSCLC.

Given the critical challenge of drug resistance in HCC treatment, we hypothesize that elevated LRRC1 may also confer resistance to sorafenib or other VEGF inhibitors. Our results demonstrate that LRRC1 upregulates the key pro-angiogenic mediator VEGFA via the USP7/PDK1/AKT/GSK3β/β-catenin signaling pathway, promoting angiogenesis in HCC. This suggests a model in which LRRC1 activates a parallel VEGFA/VEGFR2 axis that sustains angiogenesis and tumor growth even when primary pathways—such as RAF/MEK/ERK signaling or other receptor tyrosine kinases—are inhibited by sorafenib or other drugs, ultimately leading to treatment resistance. However, the precise mechanisms involved warrant further investigation.

Angiogenesis is a complex biological process that mainly includes the proliferation, migration, and tissue formation of vascular endothelial cells [38]. A previous study has demonstrated that Scribble, the paralog of LRRC1, regulates cell migration and angiogenesis through sorting of integrin α5 [39]. In addition, knockdown of ERBIN, another member of the LAP family, could significantly impair the cell migration and tubular structure formation of HUVECs via the Smad1/5 pathway [40]. These findings demonstrate that the proteins of the LAP family may have an effect on the progression of angiogenesis. So far, no correlation between LRRC1 and tumor angiogenesis in HCC has been reported. In this study, our results firstly indicate a positive correlation between the expression levels of LRRC1 and VEGFA. As is widely accepted, VEGFA is the most extensively studied member of pro-angiogenic factors and plays an important role in angiogenesis. In malignancies, VEGFA can be synthesized by diverse types of cells, such as tumor cells or stromal cells [41]. Previous studies showed that high VEGFA levels in HCC tissues were associated with unfavorable clinical outcomes [42,43]. Here, our studies demonstrate that the overexpression of LRRC1 could markedly accelerate the production and secretion of VEGFA in HCC cells, which in turn stimulates tube formation, migration, and invasion of HUVECs. Moreover, inhibiting the expression of LRRC1 showed the opposite effects. Similarly, the CAM assay also revealed that the conditional medium from LRRC1-overexpressing HCC cells promoted the formation of new blood vessels. Furthermore, the IHC assay of the subcutaneous xenograft also revealed that LRRC1 could promote the expression levels of angiogenesis factors VEGFA and CD31, providing novel evidence that LRRC1 has a regulatory function in HCC angiogenesis in vitro and in vivo. These findings extend the function of the proteins of the LAP family in tumor angiogenesis and suggest LRRC1 might serve as a potential biomarker for anti-angiogenic therapy of HCC.

It is well known that the PI3K/AKT signaling pathway is involved in many cellular processes, which can activate many different effectors and play a crucial role in multiple cancers [44,45]. Especially, the phosphorylation of GSK3β, the downstream target of the active AKT, could promote the nuclear accumulation of β-catenin, thereby regulating cell apoptosis [46], stemness [47], fibrosis [48], and tumor angiogenesis [49]. Moreover, upregulation of LRRC1 in acute myeloid leukemia cells could activate the β-catenin/c-Myc axis, thereby promoting the proliferation and glycolysis [20]. In line with these previous studies, our studies showed that LRRC1 could positively regulate the protein levels of p-AKT1, p-GSK3β, and β-catenin. Furthermore, the IHC assay showed that the elevated LRRC1 promoted the expression of β-catenin in the nucleus, suggesting the effect of LRRC1 on the AKT/GSK3β/β-catenin axis. Previous research demonstrated that ginsenoside Rb1 could stimulate the production of VEGFA via the AKT/GSK3β signaling pathway [28]. In addition, VEGFA has been identified as a downstream target of β-catenin [50,51]. Similar findings were also obtained in our study; the inhibition of p-AKT1, p-GSK3β, β-catenin, and VEGFA levels induced by silencing AKT1 was partially rescued after the overexpression of LRRC1, which confirmed that LRRC1 also regulates the expression of VEGFA via the AKT/GSK3β/β-catenin axis.

**Figure 7:**
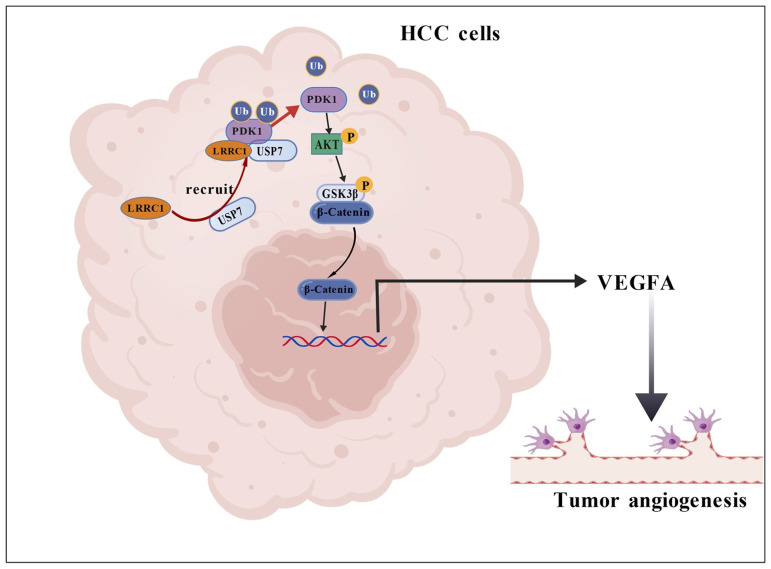
Proposed mechanism by which LRRC1 promotes angiogenesis in HCC.

Notably, PDK1 is a well-characterized upstream activator of AKT signaling and is implicated in various tumors. It acts as a primary kinase that phosphorylates AKT in the presence of PIP3 [52]. The phosphorylated AKT will further promote signal transduction through downstream effectors such as GSK3β, contributing to cancer progression. For example, one study indicated that the PDK1/PTEN/AKT/GSK3β/CyclinD1 pathway was involved in the proliferation of keratinocytes in cholesteatoma [53]. Another report demonstrated that EV-pIgR activates PDK1/Akt/GSK3β/β-catenin signaling cascades to promote HCC stemness and aggressiveness [54]. Consistent with these findings, we found that overexpression and inhibition of LRRC1 also increased and decreased PDK1 expression in HCC cells, respectively, confirming the regulation of LRRC1 on the PDK1/AKT/GSK3β/β-catenin signaling cascade. Furthermore, it is worth noting that USP7, a critical deubiquitinating enzyme, has been identified as a key interactor of PDK1 that stabilizes it via de-ubiquitination [55,56]. In line with this, our data confirm that endogenous USP7 binds to PDK1 in HCC cells. Specifically, we previously demonstrated that LRRC1 enhances the stability of p-Smad2/3 by modulating ubiquitination during hepatic stellate cell activation [57], prompting us to speculate whether a similar ubiquitination-related mechanism might be involved in the present context. Notably, published mass spectrometry data suggest that LRRC1 is also a putative interactor of USP7 [31]. This interaction was experimentally validated in HCC cells via Co-IP assays in our study. Unexpectedly, additional Co-IP assays revealed a direct interaction between LRRC1 and PDK1, indicating that LRRC1, PDK1, and USP7 form a ternary complex. As reported, LRRC1 functions as a scaffold protein with important roles in cellular development and apicobasal polarity [17,58]. Structurally, it contains leucine-rich repeats (LRR) and PDZ-binding motifs (PDZ-BMs), which are critical for mediating protein–protein interactions. For instance, LRRC1 and Lgl2 were previously shown to interact via the LRR domains [58]. These structural and regulatory properties support a scaffold function for LRRC1. Crucially, neither overexpression nor knockdown of LRRC1 affected USP7 expression levels. Based on these results, we propose a model in which LRRC1 serves as a central scaffold that recruits USP7 to PDK1, thereby facilitating USP7-mediated de-ubiquitination of PDK1. The subsequent accumulation of PDK1 enhances AKT1 phosphorylation, activating downstream signaling events, including VEGFA upregulation, that ultimately promote angiogenesis in HCC (Figure 7, created with BioGDP.com).

However, this study has several limitations. First, although AKT1 regulates multiple downstream pathways, our research focused specifically on the GSK3β pathway, leaving the potential effects of other signaling cascades such as NF-kB and FOXO1 unexplored. Future studies should investigate these additional pathways. Second, the precise binding sites of the LRRC1-USP7-PDK1 interaction remain to be elucidated through detailed mutation or truncation analyses. Finally, as our conclusions are derived primarily from cell line models, further investigation in more physiologically relevant systems, such as patient-derived organoids or in vivo models, will be essential to confirm the pathophysiological relevance of the proposed mechanism. Despite these limitations, we believe that this does not impact the main conclusions of the study.

## 5. Conclusions

In this study, we demonstrate that LRRC1 functions as a pro-angiogenic factor that promotes HCC angiogenesis by enhancing VEGFA expression via the USP7/PDK1/AKT/GSK3β/β-catenin signaling cascade. These findings not only elucidate a previously unrecognized role and mechanism of LRRC1 in HCC progression but also highlight the LRRC1-VEGFA axis as a potential therapeutic target for HCC and other malignant solid tumors.

## Data Availability

The original contributions presented in this study are included in the article/Supplementary Material. Further inquiries can be directed to the corresponding author.

## Supplementary Materials

The following supporting information can be downloaded at: https://www.mdpi.com/article/10.3390/cells14231919/s1, Table S1: siRNA or shRNA sequences; Table S2: Primer sequences for quantitative real-time PCR analysis; Figure S1: LRRC1 was upregulated in HCC cell lines; Figure S2: LRRC1 promoted the invasion ability of HUVECs; Figure S3: LRRC1 promoted the expression of p-AKT and p-GSK3β in vivo. File S1. Original Western blot images.

## Abbreviations

The following abbreviations are used in this manuscript:

CAM	Chick chorioallantois membrane
CM	Conditioned medium
HCC	Hepatocellular carcinoma
IHC	Immunohistochemistry
LAP	Leucine-rich repeat and PDZ domain
LRRC1	Leucine-rich repeat-containing 1
NSCLC	Non-small cell lung cancer
OS	Overall survival
PFS	Progression-free survival
VEGF	Vascular endothelial growth factor
