# Supplementary material for: LRRC1 Promotes Angiogenesis Through Regulating AKT/GSK3β/β-Catenin/VEGFA Signaling Pathway in Hepatocellular Carcinoma
Source: Cells. 2025 Dec 3;14(23):1919. doi: 10.3390/cells14231919 (PMC12691226; doi:10.3390/cells14231919)
Supplement: Supplementary file 1 [file cells-14-01919-s001.zip › cells-3971775-supplementary.pdf]

Supplementary materials for “LRRC1 promotes angiogenesis through regulating AKT/GSK3β/β-catenin/VEGFA signaling pathway in hepatocellular carcinoma”

Supplementary Tables

Table S1 | siRNA or shRNA sequences

Gene name	sequence (5’-3’)
sh-LRRC1	CTAGAGGAACTGAGAGAGAATCTTCTCTTTCCTTTTT
	CGAGAGAAGATTCTCTCTCAGGA
si-AKT1	GGAGGGUUGGCUGCACAAATT

Table S2 | Primer sequences for quantitative real-time PCR analysis

Gene name	Primer sequence (5’-3’)
h-GAPDH-F	GCACAGTCAAGGCCGAGAAT
h-GAPDH-R	GCCTTCTCCATGGTGGTGAA
h-LRRC1-F	TCCTTACCAAAAGAGATCGG
h-LRRC1-R	GGTAGATGCAGCAACCTGT
h-VEGFA-F	ATCGAGTACATCTTCAAGCCAT
h-VEGFA-R	GTGAGGTTTGATCCGCATAATC

Supplementary Figures

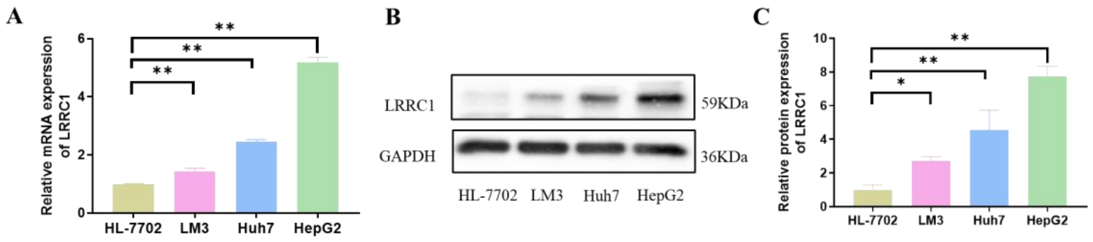

Figure S1 | LRRC1 was upregulated in HCC cell lines. (A) The mRNA expression of LRRC1 was detected in human HCC cell lines including HCC-LM3, Huh7, HepG2 cells and normal liver cells HL-7702 cells. (B) Western-blot analysis of LRRC1 expression in human HCC cell lines including HCC-LM3, Huh7, HepG2 cells and normal liver cells HL-7702 cells. (C) Quantitative results of western blot. Data represented as the mean ± SD ($^{*}P < 0.05$, $^{**}P < 0.01$).

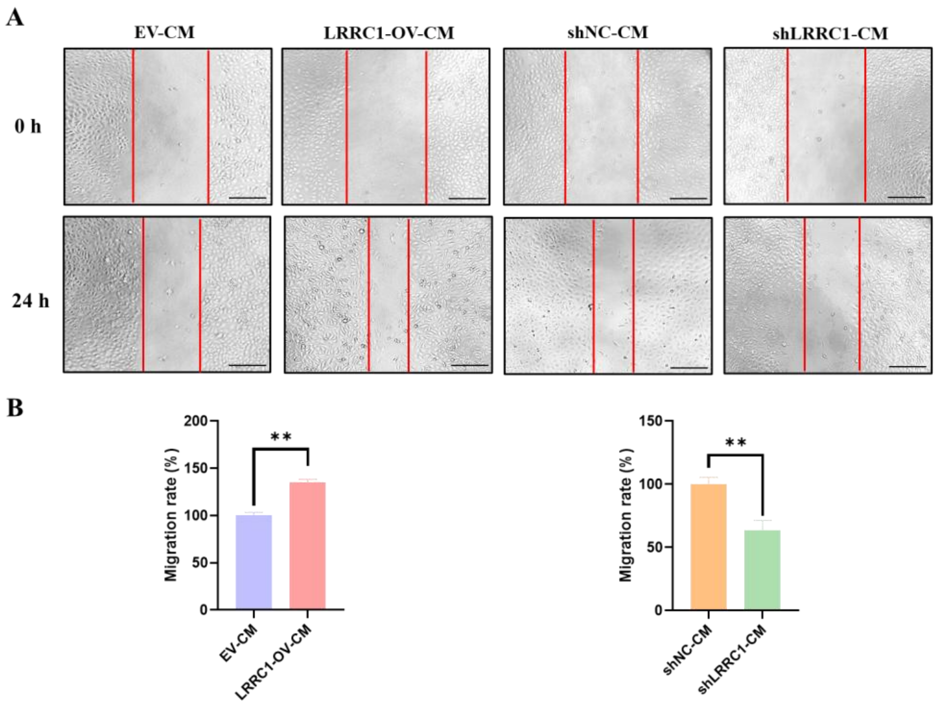

Figure S2 | LRRC1 promoted the invasion ability of HUVECs. (A, B) Representative images (A) and quantification (B) of migration cells of HUVECs treated with conditioned medium (CM) collected respectively from the LRRC1-overexpressing HCC-LM3 cells (LRRC1-OV), corresponding control cells (EV), LRRC1-knockdown HepG2 cells (shLRRC1), and the corresponding control cells (shNC) for 24h (n=3). Scale bars, 200 μm. Data represented as the mean ± SD ($^{**}P < 0.01$).

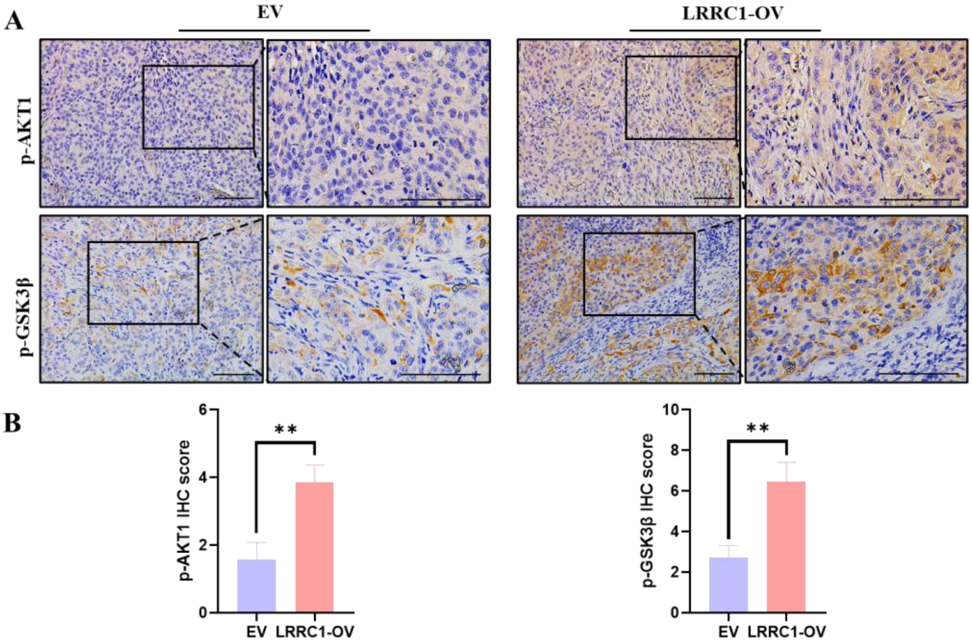

Figure S3 | LRRC1 promoted the expression of p-AKT and p-GSK3β *in vivo*. (A, B) Representative images of IHC staining (A) and analysis (B) of the expression levels of p-AKT and p-GSK3β in xenografted tumor tissues from EV group and LRRC1-OV group (n=3). Scale bars, 100 μm. Data represented as the mean ± SD ($^{**}P < 0.01$).
